# *Streptococcus agalactiae* Infection in Wild Trahira (*Hoplias malabaricus*) and Farmed Arapaima (*Arapaima gigas*) in Brazil: An Interspecies Transmission in Aquatic Environments Shared with Nile Tilapia (*Oreochromis niloticus*)

**DOI:** 10.3390/microorganisms12122393

**Published:** 2024-11-22

**Authors:** Carlos Augusto Gomes Leal, Rafael Gariglio Clark Xavier, Guilherme Alves de Queiroz, Tarcísio Martins França Silva, Júnia Pacheco Teixeira, Flávia Figueira Aburjaile, Guilherme Campos Tavares

**Affiliations:** Department of Preventive Veterinary Medicine, Veterinary School, Federal University of Minas Gerais, Belo Horizonte 31270-901, Brazil; rafaelgariglio90@gmail.com (R.G.C.X.); guiaqua7@yahoo.com.br (G.A.d.Q.); tarcisiomfes@gmail.com (T.M.F.S.); junia_pacheco@yahoo.com.br (J.P.T.); faburjaile@gmail.com (F.F.A.); gcamposvet@hotmail.com (G.C.T.)

**Keywords:** antimicrobial resistance, bacteria, fish, genetic diversity, genomic analysis

## Abstract

*Streptococcus agalactiae* is an important pathogen responsible for cases of high mortality in farmed and wild fish worldwide. In Brazil, this bacterium has been commonly associated with outbreaks in Nile tilapia farms, but other native fish species are also susceptible. Since floating cages are one of the most common culture systems used in the country, the close contact between farmed tilapia and native fish species presents a risk concerning the transmission of this pathogen. In this study, we characterized a mortality outbreak in free-living trahira and in farmed arapaima, as well as the genetic and antimicrobial susceptibility patterns of the isolates obtained. During the outbreaks, moribund fish were sampled and subjected to bacterial examination, after which the isolates were identified via MALDI-ToF analysis. Genotyping was evaluated using repetitive sequence-based PCR (REP-PCR) and multilocus sequence typing (MLST). Antimicrobial susceptibility was evaluated using disc diffusion assays. In addition, whole-genome analysis also was performed. *S. agalactiae* was identified in all diseased fish, all of which belonged to serotype Ib; however, trahira strains were classified as non-typeable lineages in the MLST assay, while arapaima strains were classified as ST260. These isolates were shown to be similar to the main genotype found in Nile tilapia in Brazil, using REP-PCR, MLST and phylogenomic analysis. The pathogenicity of the bacterium was confirmed by Koch’s postulates for both fish species. The antimicrobial susceptibility assay showed variable results to the same antibiotics among the isolates, prompting four of the isolates to be classified as multidrug-resistant. This study represents the first report of a natural outbreak of *Streptococcus agalactiae* infection in wild trahira and farmed arapaima inhabiting the same aquatic environment as Nile tilapia.

## 1. Introduction

*Streptococcus agalactiae* infections have been described in a large number of hosts [1], especially humans [2], cattle [3], and fish [4]. This Gram-positive bacterium is considered an important pathogen for aquaculture due to the high mortality on farms worldwide and the septicemia and meningoencephalitis caused in a wide range of fish species, including captive and wild fish, with tilapia and its hybrids the most affected [5,6]. In Brazil, this pathogen has been isolated from the Nile tilapia (*Oreochromis niloticus*), Amazon catfish (*Leiarius marmoratus* × *Pseudoplatystoma corruscans*), barred sorubim (*Pseudoplatystoma reticulatum*), prochilod (*Prochilodus lineatus*), and pearl cichlid (*Geophagus brasiliensis*) in monoculture or polyculture with tilapia [4,7,8,9]. There are no descriptions concerning the natural outbreak of *S. agalactiae* infections in free-living fish in Brazil.

Trahira (*Hoplias malabaricus*, Bloch) is a native freshwater fish species, widely distributed in the hydrographic basins of Central and South America, and found in different aquatic environments such as rivers, lakes, floodplains, and reservoirs [10,11]. This predatory fish, which is essentially piscivorous [12], has also been used as an ornamental fish [13], and its meat is much appreciated in Brazilian cuisine [11]. Thereby, there was an increase in trahira production in Brazilian aquaculture [13], especially in the southern region, where the total fish production reached 793,224 tons in 2022 [14]. Currently, the causes of disease outbreaks in trahira are unknown. The identification of parasite communities, such as protozoans, metacercariae, Digenea, nematodes, and parasitic crustaceans [15,16,17], as well as the isolation of *Aeromonas* spp. from fillets sold in a commercial establishment [18] have been reported in trahira. Nevertheless, there is still no evidence supporting that such organisms may be involved in the disease etiology of this aquatic host. Beyond this, there are no other reports concerning *S. agalactiae* infections in trahira.

Arapaima (*Arapaima gigas*, Schinz) is considered one of the largest species of freshwater scale fish in the world, reaching more than 2 m in length and weighing about 125 kg [19,20]. Popularly known as “pirarucu”, “paiche” or “arapaima”, this fish species is commonly found in lowland lentic waters of the Amazon, Araguaia-Tocantins, and Orinoco River basins such as lakes, floodplains and flooded forest [21,22,23]. Moreover, the presence of arapaima has already been confirmed in the upper Paraná River basin [24]. The species is currently present in 12 countries, being a native species in Brazil, Colombia, Ecuador, Guyana, and Peru, and introduced in Bolivia, China, Cuba, Mexico, Philippines, Singapore, Thailand and the USA [25,26,27,28]. This fish species is a carnivorous obligate air-breathing species and its commercial production has been increasing in different Brazilian states due to its high growth rate, rusticity, adaptability to commercial feed, high carcass yield, absence of thorns in the meat, resistance to the environment with high stocking densities and low concentration of oxygen dissolved, and acceptance of its meat in the consumer market [29]. These characteristics made it possible for the arapaima production in Brazilian aquaculture to reach 2028, 247 tons in 2022 [14]. However, the production of this fish species has some obstacles, especially those related to artificial reproduction, nutrition, high mortality rates in early life stages, and the occurrence of outbreaks of parasitic and infectious diseases [30,31,32]. Among the parasitic agents that cause diseases in arapaima farms, the following stand out: *Ichthyophthirius multifiliis* [33], *Dawestrema* sp. [30], *Hysterothylacium* sp. [34], and *Polyacanthorhynchus macrorhynchus* [33]. On the other hand, bacterial infections caused by *Aeromonas hydrophila* [31], *Aeromonas jandaei* [35], *Edwardsiella tarda* [36], *Salmonella enterica* subsp. *arizonae* [37], *Serratia fonticola* [38] and lactococcosis-causing bacteria [39] have already been reported in diseased arapaima. A previous study detected a higher prevalence of *Streptococcus* spp. (16.7%) in arapaima without clinical signs from Peruvian farms that reported 2% mortality for undiagnosed causes [40]. However, the authors did not determine the isolates obtained at the species level.

Therefore, this study aimed to identify the etiological agent responsible for the mortalities in free-living trahiras and farmed arapaima in Brazil. The genotype and antibiotic resistance patterns of the isolates were evaluated. In addition, whole-genome sequencing was performed for a representative strain of each fish species to elucidate interspecies transmission and detect antimicrobial resistance genes.

## 2. Materials and Methods

### 2.1. Outbreaks

#### 2.1.1. Free-Living Trahiras

In March 2017, cases of mortality were observed in free-living trahiras inhabiting a lake on a farm in the city of Mateus Leme/MG (Geographic coordinates: 19°59′23″ S, 44°25′21″ W). In this lake, there are other wild native fish species and Nile tilapia reared in floating cages. During the evaluation period, the concentration of dissolved oxygen remained above 6.0 mg/L, the water temperature varied between 28 and 30.5 °C, and eight diseased adult trahiras were observed. Of these, three moribund trahiras (average length of 50 ± 4.24 cm) were sampled and transported on ice to the laboratory for analysis. The remaining fish were found dead and in an advanced stage of autolysis. Therefore, these fish were not collected, as their inclusion could compromise the quality of the bacteriological examination. Previous cases of streptococcosis in farmed tilapia were reported by the farmer in the previous fish production cycle, approximately 8 months earlier. However, during the studied outbreak, only wild trahira presented clinical signs of diseases.

#### 2.1.2. Farmed Arapaima

Between April and July 2017, a disease outbreak occurred in a commercial fish farm located in the city of Montes Claros/MG (Geographic coordinates: 16°43′41″ S, 43°51′54″ W). On this farm, Nile tilapia and arapaima are reared in four excavated tanks, however, mortalities were verified in arapaima tanks. The total mortality during the outbreak reached 0.7% of the batch. At the time of sampling in the farm, six moribund fish (average weight of 40 ± 12 g) were identified, sampled, and transported on ice to the laboratory for analysis.

### 2.2. Bacteriological Examinations

Brain and kidney tissues were aseptically sampled, streaked onto tryptone soybean agar (Kasvi, Torrejón de Ardoz, Spain) supplemented with 5% defibrinated sheep blood (TSAB), and incubated at 28 °C for 48 h for bacterial isolation. Next, pure colonies were subjected to Gram staining, catalase and oxidase tests, and Lancefield’s serotyping using the Slidex Strepto Plus kit (BioMerieux, Craponne, France). The bacterial species were confirmed via MALDI-ToF mass spectrometry analysis using Bruker Microflex MALDI Biotyper 2.0 (Bruker Daltonics, Bremen, Germany), as previously described [41]. Then, the isolates were stored in 15% glycerol BHI (“Brain Heart Infusion”, Himedia, Mumbai, India) broth at −70 °C until further use.

### 2.3. DNA Extraction

The *Streptococcus agalactiae* isolates obtained from this study were thawed, streaked onto TSAB, and incubated at 28 °C for 48 h. Then, the bacterial DNA was extracted with the Wizard Genomic DNA Purification kit (Promega, Madison, WI, USA) according to the manufacturer’s instructions. The extracted DNA was quantified with the Nanodrop 2000 spectrophotometer (Thermo Scientific, Waltham, MA, USA) and stored at −20 °C until use as a template in the PCR assays.

### 2.4. Capsular Typing

The capsular polysaccharide typing was performed via a multiplex PCR assay, as described by Poyart et al. [42] with some modifications. The multiplex PCR was performed with a GoTaq DNA Polymerase kit (Promega, USA) and a final volume of 25 µL. The mix was composed of 5 µL of 1 × PCR buffer, 0.25 µL of each previously designed PCR primer [42], 0.5 µL of dNTPs, 2 µL of MgCl_2_, 2 U (0.4 µL) of Taq DNA polymerase, and 100 ng of template DNA. The PCR conditions consisted of an initial step of 95 °C for 15 min, followed by 35 cycles of 95 °C for 1 min, 63 °C for 1 min, and 72 °C for 1 min, with a final elongation step of 72 °C for 10 min. The primers used were acquired from Integrated DNA Technologies (IDT, Coralville, IA, USA). The multiplex PCR was performed using the Veriti 96-well Thermal Cycler equipment (Life Technologies, Carlsbad, CA, USA). The PCR products were separated via 1.5% agarose gel electrophoresis (70 V for 180 min), with the 100 bp marker (Invitrogen, Waltham, MA, USA) as the molecular size standard. The gel was stained with ethidium bromide (0.5 μg mL^−1^ for 30 min) and subsequently visualized via UV transillumination. Images were captured using the L-Pix EX equipment (Loccus Biotechnology, Cotia, Brazil). The SA07 (serotype Ia), SA16 (serotype Ib), and NEM316 (serotype III) strains were included as positive controls for the multiplex PCR. To confirm the serotype, the isolates were subjected to serotyping using the Strep B Latex Ib (batch LBIb-P1; Statens Serum Institute, København, Denmark) according to the manufacturer’s recommendations.

### 2.5. Repetitive Extragenic Palindromic-PCR (REP-PCR)

The genetic diversity of the isolates was evaluated by repetitive extragenic palindromic-PCR (REP-PCR), described by Costa et al. [43] with some modifications. In addition, *Streptococcus agalactiae* strains isolated from diseased Nile tilapia (SA52 and SA95) and Amazon catfish (SA103) were included in this analysis. These strains had the DNA extracted as described above.

PCR was performed with a HotStart Taq Polymerase kit and a final volume of 25 µL. The mix was composed of 5 µL of 1 × PCR buffer, 1.25 µL of GTG5 (5′-GTGGTGGTGGTGGTG-3′) (Integrated DNA Technologies, EUA), 0.5 µL of dNTPs, 3 µL of MgCl_2_, 0.5 µL of Taq DNA polymerase, and 100 ng of template DNA. The PCR conditions consisted of an initial step of 95 °C for 15 min, followed by 30 cycles of 95 °C for 30 s, 45 °C for 1 min, and 72 °C for 8 min, with a final elongation step of 72 °C for 16 min. The Veriti 96-Well Thermal Cycler also was used. The amplicons were separated by electrophoresis on a 2% agarose gel, stained with ethidium bromide (0.5 mg mL^−1^ for 30 min), visualized by UV transillumination, and the images were captured using an L-Pix EX. A 1 kb DNA ladder (Invitrogen, USA) was used as the molecular size standard. The REP-PCR images were analyzed using BioNumerics version 6.6 (Applied Maths, Sint-Martens-Latem, Belgium). The Dice coefficient was used to analyze the similarities of the banding patterns [44]. Dendrograms were created using the unweighted pair group method with an average (UPGMA) approach. Isolates that showed similarity ≥ 92% were considered clonally related [45]. The discriminatory power of REP-PCR was calculated using Simpson’s index of diversity (*D*) [46]. This analysis was performed using the online software Comparing Partitions version 06.10.2011 (available at http://www.comparingpartitions.info/, accessed on 8 August 2023) [47].

### 2.6. Multilocus Sequence Typing (MLST)

The MLST analysis was performed for the sequencing of the seven housekeeping genes (*adhP*, *pheS*, *atr*, *glnA*, *sdhA*, *tkt*, and *glcK*), following the methodology described by Jones et al. [48] with some modifications. The PCR was performed with a HotStart Taq Polymerase kit (Qiagen, Germantown, MD, USA) and a final volume of 25 µL. The reaction mixture and PCR conditions for each gene are shown in Table 1. All reactions were performed using the Veriti 96-well Thermal Cycler equipment. The PCR products were separated via 1.5% agarose gel electrophoresis, and stained images were captured as described above. Then, the PCR products were purified with the Purelink PCR purification kit (Invitrogen, USA), according to the manufacturer’s instructions. The gene sequencing was performed using a BigDye^TM^ Termination v3.1 Cycle sequencing kit (Applied Biosystems, Waltham, MA, USA) and ABI 3500 Genetic Analyzer (Life Technologies). Contigs were generated for each gene using the BioEdit v.7.2.0 software (Ibis Biosciences, Carlsbad, CA, USA). The ST of the isolates was determined using the *Streptococcus agalactiae* MLST database (https://pubmlst.org/organisms/streptococcus-agalactiae, accessed on 22 March 2022) [49].

### 2.7. Whole-Genome Sequencing and Phylogenomic Analysis

Whole-genome sequencing was performed on the Illumina HiSeq 2000 platform with 125 bp paired-end reads. Sequencing libraries were constructed according to the Illumina protocol using 100 ng of genomic DNA. Annotated assemblies were produced using a pipeline described by Page et al. [50]. To determine the genomic relationship between *S. agalactiae* isolates from trahira and arapaima with other isolates obtained from fish in Brazil, we conducted whole-genome sequencing of SA32-17 and SA45-17 strains and compared phylogenomically with genomes deposited on GenBank (Supplementary Table S1). Reads were assembled using VelvetOptimiser v2.2.5 (https://github.com/tseemann/VelvetOptimiser, accessed on 10 February 2022) and Velvet v1.2 [51]. SSPACE v.2.0 [52] and GapFiller v.2.1.2 [53] were used to scaffold the contigs and sequence gaps filling, respectively. Assembly quality and statistics were evaluated with QUAST v.5.2 [54]. Automated annotation was performed using PROKKA v1.11 [55] and a *Streptococcus*-specific database from RefSeq v.226 [56]. Assembled contigs were also evaluated for identification and characterization of antimicrobial resistance genes using the PanViTa tool v.1 [57].

We inferred a phylogenomic tree on the selected genomes via the codon tree method, which uses RAxML to align single-copy cross-genus protein families (PGFams). In parallel, we also inferred the degree of genomic relatedness among SA32-17, SA45-17 and other Brazilian *Streptococcus agalactiae* fish strains (Supplementary Table S1) with Average Nucleotide Identity (ANI) analysis through the MUMmer alignment method using pyANI (v3.0) [58]. Further, we used Orthofinder v.2.5.5 for tree plotting and visualization [59].

### 2.8. Antimicrobial Susceptibility

*Streptococcus agalactiae* strains were submitted to antimicrobial susceptibility tests via the disc diffusion methodology, according to the Methods for Antimicrobial Disk Susceptibility Testing of Bacteria Isolated from Aquatic Animals (VET03) guidelines, with adaptations recommended for streptococci bacteria (Group 4) [60]. The antimicrobial discs (Oxoid, Altrincham, UK) tested were oxytetracycline (OXY, 30 μg), florfenicol (FLO, 30 μg), sulfamethoxazole/trimethoprim (SXT, 25 μg), amoxicillin (AMO, 10 μg), norfloxacin (NOR, 10 μg) and erythromycin (ERY, 15 μg). The *Streptococcus agalactiae* isolates were thawed, streaked onto TSAB, and incubated at 28 °C for 48 h. Colonies of each strain were transferred to a sterile saline solution to achieve an absorbance value of 0.08–0.13 (OD_625_), as assessed using a spectrophotometer, and the bacteria were streaked onto a Müller Hinton standard agar medium (Kasvi, Italy) supplemented with 5% defibrinated sheep blood. Subsequently, three discs of antibiotics were applied per plate. After incubation at 28 °C for 48 h, the diameters of the inhibition halos were measured with a digital caliper (Vernier Caliper). This step was performed in triplicate. The isolates were classified as wild- (susceptible) or non-wild-types (resistant), according to *Streptococcus agalactiae* epidemiological cutoff values (CO_WT_) previously calculated for isolates from fish [9,61] using normalized resistance interpretation (NRI) [62]. In addition, the criteria and standardized international terminology for defining multidrug resistance (MDR) created by the European Centre for Disease Prevention and Control (ECDC) and the Centers for Disease Control and Prevention (CDC) were followed [63]. MDR was defined as an isolate that was non-susceptible to at least one agent in three or more antimicrobial categories.

### 2.9. Fish and Challenge Assay

To fulfill Koch’s postulate, trahira (average length of 13.8 ± 1.1 cm) and arapaima (19.09 ± 1.49 cm) juveniles that had previously been conditioned to the acceptance of dried food were donated from a commercial hatchery with no disease antecedents. Upon arrival at the laboratory, four fish of each species were randomly collected and submitted for bacteriological examinations, as previously described, to confirm the lack of bacterial infection at the onset of the experiment period. Fishes were acclimated in two 57 L aquaria (covered with black plastic to keep the animals under low luminosity) supplied with flow-through dechlorinated tap water (0.5 L h^−1^) at 28 °C for 15 d. The fish were subject to a 12:12 h light/dark photoperiod and were fed to satiation with Nutripiscis 42% PB (Presence, Brazil) twice a day. One *Streptococcus agalactiae* strain obtained of each host was randomly selected for the challenge assay.

For trahira infection, the SA32-17 strain was inoculated in BHI broth and incubated at 28 °C for approximately 20 h under low agitation until a bacterial concentration of 1.19 × 10^6^ CFU mL^−1^ was reached. Before the challenge assay, fish were anesthetized by immersion in a benzocaine solution (100 mg L^−1^) (Sigma-Aldrich, St. Louis, MO, USA). Four fish were treated with intraperitoneal injections (infected group, G1) with 0.1 mL of SA32-17 inoculum (bacteria in BHI broth) corresponding to a dose of 10^5^ CFU fish^−1^, whereas two fish (control group, G2) were injected with 0.1 mL of sterile BHI.

For arapaima infection, the SA45-17 strain was selected. This isolate was inoculated in BHI broth and incubated at 28 °C for approximately 19 h under low agitation until a bacterial concentration of 5.2 × 10^7^ CFU mL^−1^ was reached. Before the challenge assay, fish were also anesthetized by immersion in a benzocaine solution (100 mg L^−1^). Eight fish were treated with intraperitoneal injections (infected group, G3) with 0.1 mL of SA45-17 inoculum corresponding to a dose of 10^6^ CFU fish^−1^, whereas eight (control group, G4) were injected with 0.1 mL of sterile BHI.

The infected fish (G1 and G3) and control groups (G2 and G4) were monitored and fed twice a day for 14 d. Dead fish were removed and subjected to bacterial isolation from the sampling of brain and kidney tissues. These tissues were aseptically collected, streaked onto TSAB, and incubated at 28 °C for 72 h. Then, the isolates were identified via MALDI-ToF, as previously described. The same procedure was performed in all fish that remained alive at the end of the experimental period after euthanasia with a benzocaine overdose (300 mg L^−1^) for the detection of carrier fish. This in vivo experiment was approved by the Ethics Committee for Animal Use of the Federal University of Minas Gerais (CEUA-UFMG—316/2019 and 143/2021).

### 2.10. Histopathological Analysis

Fragments of the brain, kidney, liver, spleen, and heart were collected for histopathological analysis. The tissues collected from all fish of infected and control groups were fixed in neutral buffered 10% formalin for 24 h. Next, the tissues were dehydrated through immersion in ethanol solutions of increasing concentrations (70–100%), cleared with xylene, and embedded in paraffin using a vacuum infiltration processor (TP 1020, Leica Biosystems, Nußloch, Germany). Tissues were sectioned in paraffin blocks at 4 µm using the semi-automated rotary microtome (RM2245, Leica Biosystems, Singapore) and stained with hematoxylin-eosin (HE) [64]. Stained sections were examined using light microscopy (Olympus BX41, Olympus, Tokio, Japan) and photographed using an automated upright microscope system (Leica DM4000 B, Leica Biosystems, Singapore).

## 3. Results

### 3.1. Clinical Signs at Field Conditions and Identification of Streptococcus Agalactiae Isolates

**Figure 1:**
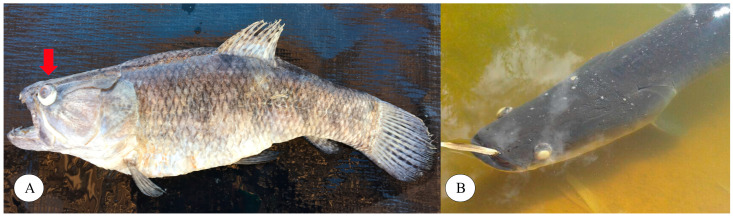
Natural *Streptococcus agalactiae* infection in native fish species. (**A**) Diseased wild trahira with exophthalmia (arrow) found dead during the outbreak; (**B**) Diseased arapaima with exophtalmia and corneal opacity, alive in the tank.

The main clinical signs verified in the diseased trahira were lethargy, exophthalmia (Figure 1A), and corneal opacity, while in diseased arapaima, anorexia, lethargy, exophthalmia, corneal opacity (Figure 1B), erratic swimming and ascites were observed.

In the bacteriological analysis, a pure culture with small, translucent, and nonhemolytic colonies was obtained from all tissues sampled from the analyzed fish. The isolates were characterized as Gram-positive cocci arranged in chains, negative in catalase and oxidase tests, and classified as Lancefield Group B. Bacterial identification using MALDI-ToF confirmed that *Streptococcus agalactiae* was the etiological agent associated with the disease outbreak in the wild trahira and farmed arapaima.

### 3.2. Genetic Diversity and Relationship Among the Isolates

All *Streptococcus agalactiae* isolates (SA31, SA32, SA33, SA45, SA100, SA108, SA110, SA112 and SA174—strain codes from culture collection; one strain from each fish analyzed) obtained belonged to serotype Ib by multiplex PCR and confirmed conventional serotyping with Strep B Latex Ib.

**Figure 2:**
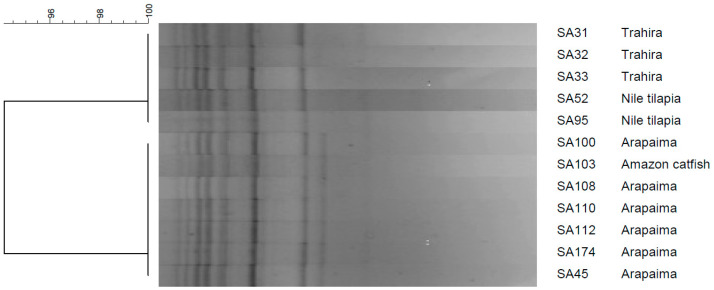
REP-PCR results for five *Streptococcus agalactiae* strains isolated from trahira, arapaima, Amazon catfish and Nile tilapia. The dendrogram was constructed using Dice’s coefficient and the UPGMA method.

REP-PCR of the isolates resulted in the amplification of eight bands, ranging in size from 500 to 5000 bp. A single REP profile was detected, with a similarity of 94% (*D* = 1.0), indicating that the isolates obtained from trahira, arapaima, Amazon catfish and Nile tilapia are clonally related (Figure 2).

The MLST analysis of *Streptococcus agalactiae* strains obtained from trahira showed positive PCR results for six of the seven genes tested, corresponding to allelic profiles *adhP*: 52; *atr*: 31; *glnA*: 4; *pheS*: 17; *sdhA*: 26; and *tkt*: 19. Negative results were obtained for the *glcK* gene. The occurrence of a partially deleted *glcK* gene was previously characterized in *Streptococcus agalactiae* isolates from fish in Brazil [65]. In this manner, these isolates cannot be categorized into an ST group, and are instead determined as a non-typeable (NT) lineage. On the other hand, the isolates obtained from arapaima showed positive PCR results for all seven genes tested, corresponding to allelic profiles *adhP*: 52; *atr*: 31; *glnA*: 28; *pheS*: 17; *sdhA*: 26; *glcK*: 26 and *tkt*: 19. These isolates were categorized into ST-260.

### 3.3. Genomic Similarity and Phylogenomic Analysis

A summary of the genomic features of SA32-17 and SA45-17 is provided in Table 2. The genome project has been deposited in the GenBank database under BioProject accession number PRJNA1179504.

**Figure 3:**
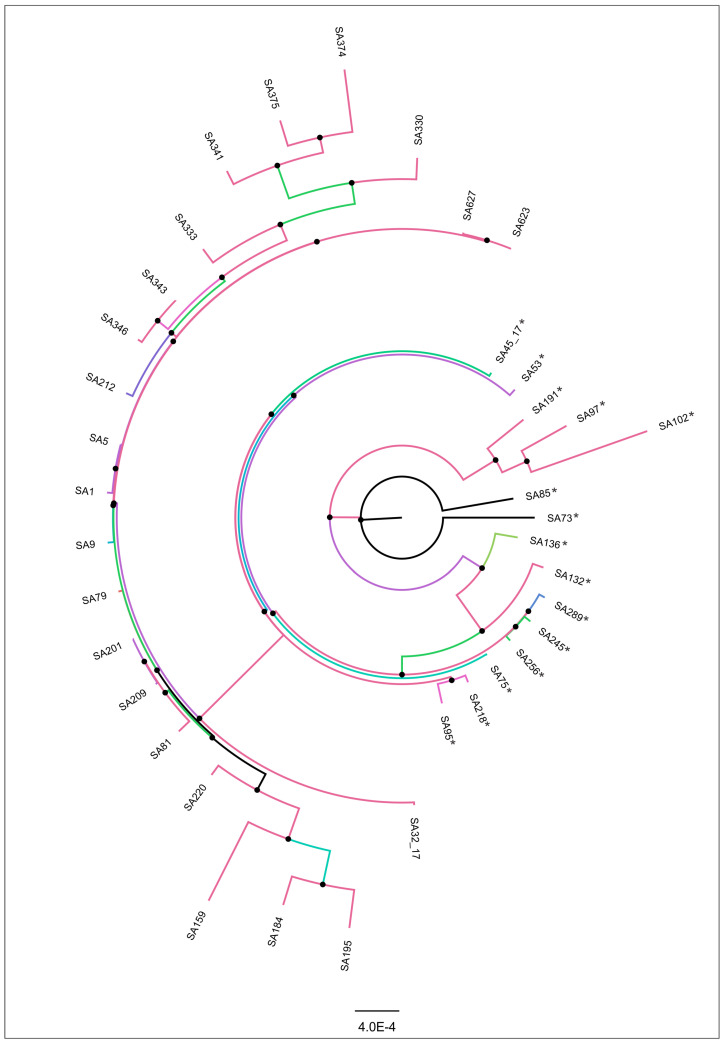
Whole-genome sequence RA × ML phylogenetic tree constructed using SA32-17 and SA45-17 strains together other Brazilian *Streptococcus agalactiae* strains deposited in the GenBank database (bootstrap test, 1000 replicates). Two distantly related groups of isolates are visualized one on the right side (CC260, marked with *) and one on the left side (NT) of the figure. Bootstrap values are presented as color gradients at the branches for better visualization.

The ANI analysis showed that SA32-17 and SA45-17 genomes were >99% identical to other piscine *Streptococcus agalactiae* strains. Although the isolates showed a high similarity, in the phylogenomic analysis, it is possible to observe the formation of two slightly distinct groups, one constituted with NT strains and the other with CC260 (ST260 and ST927) strains. SA32-17 strain grouped with NT and SA45-17 with CC260 strains in accordance with the MLST results (Figure 3).

### 3.4. Antimicrobial Susceptibility and In Silico Identification of AMR Genes

In the disc diffusion assay, *Streptococcus agalactiae* strains exhibited zones ranging between 6 and 32 mm (Table 3). SA32, SA100, SA108, SA112 and SA174 strains were classified as MDR; nevertheless, they showed non-wild-type phenotypes to three or four different categories of antibiotics. All isolates showed a wild-type phenotype to OXY and FLO, and non-wild-type to SXT. A total of 33.3%, 33.3% and 66.7% of isolates showed wild-type phenotype to AMO, NOR and ERY, respectively (Table 3).

A search on the genome sequences of SA32-17 and SA45-17 strains for AMR genes detected genes that suggest the fluoroquinolone (*arlR*, *norB*, *pmrA*), macrolide (*RlmA(II)*, *efrA*, *mreA*), lincosamide (*lmrD* and *lmrP*), rifamycin (*rpoB2*) antibiotics resistance, and peptide antibiotic (*Saga_mprF*). *QacJ*, related to disinfecting agents and antiseptics resistance, was exclusively detected in the SA32 strain, while *efrB*, related to macrolide resistance, was detected just in the SA45 strain (Table 2).

### 3.5. Challenge Assay and Histopathological Analysis

**Figure 4:**
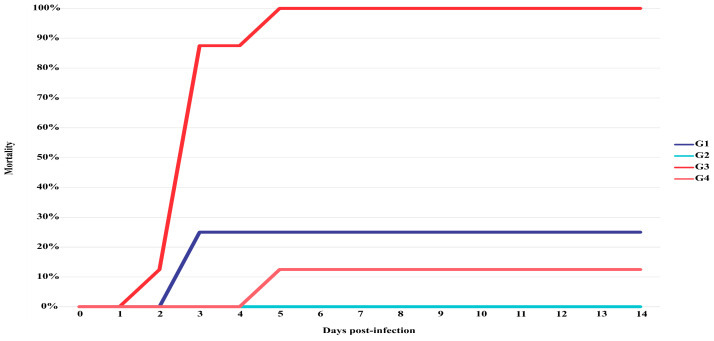
Mortality rate of the trahira and arapaima challenged with *Streptococcus agalactiae* during the experimental period.

**Figure 5:**
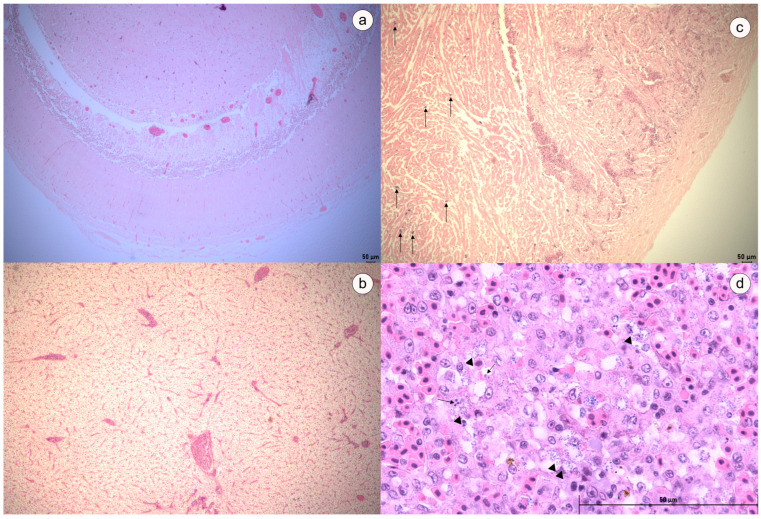
Histology of challenged trahira (**a**–**c**) and arapaima (**d**). (**a**) Brain—meningitis was and obvious sign characterized by thickening with congested blood vessels and inflammatory cells infiltration; (**b**) Liver—congestion and thrombosis of portal blood vessel accompanied with hepatic sinusoids with marked vacuolar degeneration change of hepatocytes; (**c**) Heart—Accumulation of inflammatory cells (arrows) and hemorrhagic areas in myocardium and pericardium; (**d**) Spleen—cellular necrosis (picnosis = arrow) in white pulp associated with the presence of Gram-positive bacteria (arrow head).

Clinical diseases caused by *Streptococcus agalactiae* were successfully reproduced under experimental conditions (Groups 1 and 3). Anorexia, pallor of the integument, buccal hyperemia, and corneal opacity were the main clinical signs observed in infected trahira. Only one instance of mortality was observed in the infected group (72 h post-infection) (Figure 4). However, positive bacterial recovery was verified in all remaining infected fish. During the necropsy, two of the three fish that remained alive showed signs of renal congestion. Neither clinical signs nor mortalities were observed in the control group (Group 2). In histopathological analysis, the trahira of the control group showed normal tissue architecture. However, the infected fish exhibited meningitis (Figure 5a), inflammatory cell infiltration in brain and heart tissues, congestion and thrombosis of blood vessels in brain and liver tissues, vacuolar degeneration of hepatocytes (Figure 5b) and hemorrhagic areas in myocardium and pericardium (Figure 5c).

On the other hand, mortality was observed in arapaima infected with *Streptococcus agalactiae* (Group 3) on the second day post-infection (dpi). In this group, the mortality rate of 100% was achieved after five days dpi (Figure 4), with positive bacterial recovery from all dead fish. Clinical signs such as anorexia, lethargy and fish at the bottom of the aquarium were verified in fish at approximately 48 h dpi. After 96 h dpi, anorexia, corneal opacity, loss of equilibrium, depigmentation of the skin and hyperemia at the bases of the pectoral fins also observed in this group. The post mortem examination revealed liver pallor. No clinical signs were observed in the control group; however, at the 5th dpi, one fish diad. A negative bacteriological result was obtained from this animal. No other mortality was observed in the control group until the end of the experimental period. In histopathology, cellular necrosis associated with the presence of Gram-positive bacteria was observed in the spleen of challenged arapaima (Figure 5d).

## 4. Discussion

This study represents the first report concerning a natural outbreak of *Streptococcus agalactiae* infection in wild trahira and farmed arapaima that shared the same aquatic environment with Nile tilapia. The clinical manifestation of the disease in the field and laboratory conditions were compatible with *Streptococcus agalactiae* infection. Anorexia, lethargy, exophthalmia, corneal opacity, and hyperemia are clinical signs commonly visualized due to this disease [4,6] and were observed in moribund fishes. Pathological changes in the brain, liver, heart and spleen tissues of infected trahira and arapaima were compatible with histopathological findings in other fish infected with *Streptococcus agalactiae* [66,67,68].

*Streptococcus agalactiae* has regularly been isolated from farmed Nile tilapia during outbreaks, mainly during the summer season [4], that correspond to December to March in Brazil. Recently, this pathogen has expanded its host spectrum to other native species reared in Brazil. Tavares et al. [9] described streptococcosis outbreaks caused by *Streptococcus agalactiae* and other streptococci in farmed Amazon catfish (*Leiarius marmoratus* × *Pseudoplatystoma reticulatum*). Similar to this study, in Tavares et al. [9], the fish were in close contact (reared in the same culture) with Nile tilapia. Based on the previous and present results, the main risk for the interspecies transmission of *Streptococcus agalactiae* in Brazil seems to be the close contact, through the aquatic environment, or co-cultivation of Nile tilapia (the main *Streptococcus agalactiae* host in Brazil) and native species. This raises two important concerns. First, farms that adopt polyculture systems between Nile tilapia and native species could create an opportunity for the interspecies adaptation and transmission of *Streptococcus agalactiae*, which is an important health issue. Second, since aquaculture expansion in Brazil has been based on the use of floating cages in lakes or dam reservoirs, this could allow the transmission of *Streptococcus agalactiae* to wild fish. As arapaima and trahira are carnivorous—particularly in the case of arapaima, where tilapia has been used as a forage species in captive feeding [69,70], a common practice mainly among breeders, and in the case of trahiras, where sharing the same aquatic environment facilitates the capture of tilapia that may escape from the tanks—the oral transmission hypothesis is suggested. Some studies have indicated that the main route of *Streptococcus agalactiae* entry in fish is through the gastrointestinal tract via ingestion of contaminated water or infected fish [5,71], which corroborates our hypothesis.

*Streptococcus agalactiae* isolates from trahira were classified as serotype Ib and non-typeable. This genetic pattern has commonly been identified in diseased Nile tilapia reared in the south-central region of Brazil, including the Minas Gerais state [65], where a disease outbreak in free-living trahira occurred. On the other hand, the isolates from arapaima were also classified as serotype Ib, but ST260. This genetic pattern has been identified in the northeast region of Brazil and Minas Gerais state [65], as well as in Nile tilapia. In this manner, the identification of the genotypes obtained in this study, regardless of the molecular typing method (MLST and REP-PCR), is similar to the strains obtained from streptococcal infections in Brazilian tilapia farms, which suggests transmission between tilapia and trahira or tilapia and arapaima. This hypothesis was corroborated by the genomic analyses of our sequenced strains when compared with other *Streptococcus agalactiae* strain isolates from Nile tilapia in Brazil. The high sequence similarity (>99%) of *Streptococcus agalactiae* strains obtained from trahira, arapaima and tilapia suggests that transmission between these hosts is likely.

*Streptococcus agalactiae* strains presented wild-type phenotypes to the tested antibiotics, with four isolates considered as MDR (SA32, SA100, SA108 and SA174). Moreover, the identification of fluoroquinolone and macrolide resistance genes, in genomic analysis, does not corroborate the disk diffusion result of SA32 and SA45 strains for norfloxacin and erythromycin, respectively. The phenomenon of multi-resistance has previously been described in *Streptococcus agalactiae* isolated from fish [9,72]. All isolates were classified as wild-types to florfenicol and oxytetracycline, and both antibiotics were approved for use in Brazilian aquaculture establishments for the treatment of *Streptococcus agalactiae* but had demonstrated therapeutic failures concerning disease control in Nile tilapia in field and laboratory conditions [73,74]. The susceptibility to the two antibiotics suggests a possible way of treating the disease for these aquatic hosts, but a future study should be carried out to prove this efficiency.

## 5. Conclusions

In conclusion, this is the first report of mortalities caused by *Streptococcus agalactiae* in free-living trahira and in farmed arapaima, in which the isolates belonged to the same genetic pattern commonly identified in Nile tilapia in Brazil. This may suggest that the commercial production of tilapia in the same aquatic environment as other native fish species may enable the adaptation of *Streptococcus agalactiae* to other host fish.

## Figures and Tables

**Table 1:** PCR mix composition (final reaction volume of 25 μL) and thermal cycling conditions used in the MLST analysis of *Streptococcus agalactiae*.

Genes	Reaction Mixture	PCR Conditions
*atr* *glnA* *tkt*	Buffer 10X: 2.5 μL 25 mM MgCl_2_: 1.5 μL 10 mM dNTP: 0.5 μL 10 mM each primer: 0.5 μL Taq DNA Polymerase: 0.25 μL	Initial step: 95 °C for 5 min Cycling: 35 cycles of 95 °C for 1 min, 56 °C for 45 s, and 72 °C for 1 min; Final elongation: 72 °C for 15 min
*glcK* *pheS*	Buffer 10X: 2.5 μL 25 mM MgCl_2_: 1.5 μL 10 mM dNTP: 0.5 μL 10 mM each primer: 0.5 μL Taq DNA Polymerase: 0.25 μL	Initial step: 95 °C for 15 min Cycling: 35 cycles of 95 °C for 1 min, 56 °C for 1 min, and 72 °C for 1 min; Final elongation: 72 °C for 10 min
*adhP* *sdhA*	Buffer 10X: 2.5 μL 25 mM MgCl_2_: 1.0 μL 10 mM dNTP: 0.25 μL 10 mM each primer: 0.2 μL Taq DNA Polymerase: 0.05 μL	Initial step: 95 °C for 5 min Cycling: 35 cycles of 95 °C for 1 min, 56 °C for 40 s, and 72 °C for 1 min; Final elongation: 72 °C for 10 min

**Table 2:** Main genomic features of SA32-17 and SA45-17 strains.

	SA32-17	SA45-17
Genome Statistics by QUAST Tool		
# contigs (≥0 bp)	29	23
# contigs (≥1000 bp)	28	23
Total length (≥0 bp)	1,824,566	1,817,806
Total length (≥1000 bp)	1,823,865	1,817,806
# contigs	29	23
Largest contig	368,081	283,920
Total length	1,824,566	1,817,806
G + C (%)	35.34	35.32
N50	136,190	124,698
N90	42,369	56,228
auN	192,272.4	144,941.0
L50	4	5
L90	13	15
N’s per 100 kbp	1.32	0.00
Attributes		
Genes (total)	1957	1922
Coding sequences (total)	1852	1834
Pseudogenes	29	26
rRNA	11	10
tRNA	64	51
tmRNA	1	1
AMR gene detection using PanViTa tool		
*arlR* (%)	50.4	52.2
*RlmA(II)* (%)	53.2	53.2
*lmrD* (%)	61.2	61.2
*efrA* (%)	52.8	52.6
*norB* (%)	59.7	59.5
*pmrA* (%)	57.5	57.3
*mreA* (%)	99.7	99.4
*efrB* (%)	0.0	53.3
*lmrP* (%)	54.4	54.4
*Saga_mprF* (%)	99.4	99.1
*rpoB2* (%)	52.2	52.2
*qacJ* (%)	58.9	0.0

**Table 3:** Disc diffusion results of GBS strains from diseased trahira. The inhibition zone diameters (mm) of each strain were measured after growth in Muller–Hinton agar enriched with 5% defibrinated sheep blood at 28 °C for 48 h. WT (wild type) and NWT (non-wild type), considered susceptible and resistant, respectively.

	Antimicrobial Agent *					
Strain ID	SXT ^a^	OXY ^b^	FLO ^b^	AMO ^b^	NOR ^a^	ERY ^a^
SA31	20 (NWT)	28 (WT)	30 (WT)	28 (NWT)	30 (WT)	25 (WT)
SA32	19 (NWT)	28 (WT)	30 (WT)	28 (NWT)	20 (WT)	17 (NWT)
SA33	18 (NWT)	30 (WT)	32 (WT)	32 (WT)	10 (NWT)	32 (WT)
SA45	6 (NWT)	25 (WT)	24 (WT)	27 (NWT)	16 (WT)	27 (WT)
SA100	6 (NWT)	27 (WT)	25 (WT)	27 (NWT)	6 (NWT)	27 (WT)
SA108	6 (NWT)	25 (WT)	26 (WT)	31 (WT)	6 (NWT)	24 (NWT)
SA110	6 (NWT)	23 (WT)	26 (WT)	34 (WT)	6 (NWT)	27 (WT)
SA112	6 (NWT)	25 (WT)	25 (WT)	27 (NWT)	6 (NWT)	28 (WT)
SA174	6 (NWT)	25 (WT)	24 (WT)	28 (NWT)	6 (NWT)	22 (NWT)

* SXT = trimethoprim-sulfamethoxazole, 25 μg; OXY = oxytetracycline, 30 μg; FLO = florfenicol, 30 μg; AMO = amoxicillin, 10 μg; NOR = norfloxacin, 10 μg; and erythromicyn, 15 μg. ^a^ cut-off based in Tavares et al. [9]; ^b^ cut-off based in Leal et al. [61].; Gray boxes represent NWT isolates.

## Data Availability

The genome sequences of the *Streptococcus agalactiae* isolated from trahira and arapaima were included in the NCBI database under BioProject accession number PRJNA1179504.

## Supplementary Materials

The following supporting information can be downloaded at: https://www.mdpi.com/article/10.3390/microorganisms12122393/s1, Table S1: Strains, host, origin, and ST of *Streptococcus agalactiae* strains used in phylogenomic analysis.

## References

[1] Lusiastuti A.M., Textor M., Seeger H., Akineden Ö., Zschöck M. (2014). The Occurrence of Streptococcus agalactiae Sequence Type 261 from Fish Disease Outbreaks of Tilapia *Oreochromis niloticus* in Indonesia. Aquac. Res..

[2] Johri A.K., Paoletti L.C., Glaser P., Dua M., Sharma P.K., Grandi G., Rappuoli R. (2006). Group B *Streptococcus*: Global Incidence and Vaccine Development. Nat. Rev. Microbiol..

[3] Keefe G.P. (1997). Streptococcus agalactiae Mastitis: A Review. Can. Vet. J. Rev. Vet. Can..

[4] Mian G.F., Godoy D.T., Leal C.A.G., Yuhara T.Y., Costa G.M., Figueiredo H.C.P. (2009). Aspects of the Natural History and Virulence of *S. agalactiae* Infection in Nile Tilapia. Vet. Microbiol..

[5] Bowater R.O., Forbes-Faulkner J., Anderson I.G., Condon K., Robinson B., Kong F., Gilbert G.L., Reynolds A., Hyland S., McPherson G. (2012). Natural Outbreak of *Streptococcus agalactiae* (GBS) Infection in Wild Giant Queensland Grouper, *Epinephelus lanceolatus* (Bloch), and Other Wild Fish in Northern Queensland, Australia. J. Fish. Dis..

[6] Evans J.J., Klesius P.H., Gilbert P.M., Shoemaker C.A., Al Sarawi M.A., Landsberg J. (2002). Characterization of B-Haemolytic Group B *Streptococcus agalactiae* in Cultured Seabream, *Sparus auratus* L., and Wild Mullet, *Liza klunzingeri* (Day), in Kuwait. J. Fish. Biol..

[7] Leira M.H., Botelho H.A., Lago A.A., De Freitas R.T.F., Garcia A.M., De Azevedo Soares Dos Santos H.C. (2019). Identification of Pathogens in Fish Polyculture Systems in Southern Minas Gerais, Brazil. Acta Vet. Bras..

[8] Sebastião F.A., Furlan L.R., Hashimoto D.T., Pilarski F. (2015). Identification of Bacterial Fish Pathogens in Brazil by Direct Colony PCR and 16S rRNA Gene Sequencing. Adv. Microbiol..

[9] Tavares G.C., de Queiroz G.A., Assis G.B.N., Leibowitz M.P., Teixeira J.P., Figueiredo H.C.P., Leal C.A.G. (2018). Disease Outbreaks in Farmed Amazon Catfish (*Leiarius marmoratus* x *Pseudoplatystoma corruscans*) Caused by *Streptococcus agalactiae*, *S. iniae*, and *S. dysgalactiae*. Aquaculture.

[10] Bialetzki A., Nakatani K., Sanches P.V., Baumgartner G. (2002). Spatial and Temporal Distribution of Larvae and Juveniles of *Hoplias malabaricus* (Characiformes, Erythrinidae) in the Upper Paraná River Floodplain, Brazil. Braz. J. Biol..

[11] da Silva C.A., Oba E.T., Ramsdorf W.A., Magalhães V.F., Cestari M.M., Oliveira Ribeiro C.A., Silva de Assis H.C. (2011). First Report about Saxitoxins in Freshwater Fish *Hoplias malabaricus* through Trophic Exposure. Toxicon.

[12] Rodrigues L.C., Santos A.C.G., Ferreira E.M., Teófilo T.S., Pereira D.M., Costa F.N. (2017). Aspectos Parasitológicos Da Traíra (*Hoplias malabaricus*) Proveniente Da Cidade de São Bento, MA. Arq. Bras. Med. Vet. Zootec..

[13] Benigno R.N.M., Knoff M., Matos E.R., Gomes D.C., Pinto R.M., Clemente S.C.S. (2014). Morphological Aspects of Clinostomidae Metacercariae (Trematoda: Digenea) in *Hoplerytrinus unitaeniatus* and *Hoplias malabaricus* (Pisces: *Erythrinidae*) of the Neotropical Region, Brazil. An. Acad. Bras. Cienc..

[14] IBGE (2023). Produção Da Pecuária Municipal.

[15] Baia R.R.J., Florentino A.C., Silva L.M.A., Tavares-Dias M. (2018). Patterns of the Parasite Communities in a Fish Assemblage of a River in the Brazilian Amazon Region. Acta Parasitol..

[16] de Oliveira Meneguetti D.U., de Oliveira Laray M.P., Camargo L.M.A. (2013). Primeiro Relato de Larvas de *Eustrongylides* Sp. (Nematoda: Dioctophymatidae) Em *Hoplias malabaricus* (Characiformes: Erythrinidae) No Estado de Rondônia, Amazônia Ocidental, Brasil. Rev. Pan Amaz. Saúde.

[17] Oliveira M.S.B., Corrêa L.L., Oliveira Ferreira D., Neves L.R., Tavares-Dias M. (2017). Records of New Localities and Hosts for Crustacean Parasites in Fish from the Eastern Amazon in Northern Brazil. J. Parasit. Dis..

[18] Guimarães L., dos Santos A.C., Ferreira E., Pereira D., Costa F. (2018). Microbiological Quality of Trahira Fish (*Hoplias malabaricus*) from Baixada Maranhense, Municipality of São Bento, MA. Arq. Inst. Biol..

[19] Honczaryk A., Inoue L.A.K.A. (2010). Anesthesia in Pirarucu by Benzocaine Sprays in the Gills. Ciência Rural..

[20] Imbiriba E.P. (2001). Potencial de Criação de Pirarucu, *Arapaima gigas*,Em Cativeiro. Acta Amaz..

[21] Fazzi-Gomes P.F., Melo N., Palheta G., Guerreiro S., Amador M., Ribeiro-Dos-santos A.K., Santos S., Hamoy I. (2017). Genetic Diversity and Differentiation in Natural Populations of *Arapaima gigas* from Lower Amazon Revealed by Microsatellites. Genet. Mol. Res..

[22] Ferraris C.J., Reis R.E., Kullander S.O., Ferraris C.J. (2003). Family Arapaimatidae (Bonytongues). Check list of the freshwater fishes of South and Central America.

[23] Pereira-Filho M., Roubach R., Baldisserotto B., de Carvalho Gomes L. (2010). Pirarucu (*Arapaima gigas*). Espécies nativas para piscicultura no Brasil.

[24] Carvalho F.R., Casatti L., Manzotti A.R., Ravazzi D.C.W. (2015). First Record of *Arapaima gigas* (Schinz, 1822) (Teleostei: Osteoglossomorpha), the “Pirarucu”, in the Upper Paraná River Basin, Southeast Brazil. Check List..

[25] Lawson L.L., Tuckett Q.M., Lawson K.M., Watson C.A., Hill J.E. (2015). Lower Lethal Temperature for Arapaima *Arapaima gigas*: Potential Implications for Culture and Establishment in Florida. N. Am. J. Aquac..

[26] Miranda-Chumacero G., Wallace R., Calderón H., Calderón G., Willink P., Guerrero M., Siles T.M., Lara K., Chuqui D. (2012). Distribution of Arapaima (*Arapaima gigas*) (Pisces: Arapaimatidae) in Bolivia: Implications in the Control and Management of a Non-Native Population. BioInvasions Rec..

[27] Ohs C., Hill J., Wright S., Giddings H.M., Durland Donahou A. (2021). Candidate Species for Florida Aquaculture: Arapaima *Arapaima gigas*. Edis.

[28] Wyman-Grothem K., Castello L., DTBS C., CRC D., ALB M., Patoka J., Stewart D., Watson C. (2024). Invasion Risk to the United States from *Arapaima* spp. Hinges on Climate Suitability. Aquac. Environ. Interact..

[29] Drumond G.V.F., de Almeida Caixeiro A.P., Tavares-Dias M., Marcon J.L., Affonso E.G. (2010). Características Bioquímicas e Hematológicas Do Pirarucu *Arapaima gigas* Schinz, 1822 (Arapaimidae) de Cultivo Semi-Intensivo Na Amazônia. Acta Amaz..

[30] da Cruz M.G., Jerônimo G.T., Bentes S.P.C., Gonçalves L.U. (2022). Trichlorfon Is Effective against *Dawestrema cycloancistrium* and Does Not Alter the Physiological Parameters of Arapaima (*Arapaima gigas*): A Large Neotropical Fish from the Amazon. J. Fish. Dis..

[31] Dias M.K.R., Sampaio L.S., Proietti-Junior A.A., Yoshioka E.T.O., Rodrigues D.P., Rodriguez A.F.R., Ribeiro R.A., Faria F.S.E.D.V., Ozório R.O.A., Tavares-Dias M. (2016). Lethal Dose and Clinical Signs of *Aeromonas hydrophila* in *Arapaima gigas* (Arapaimidae), the Giant Fish from Amazon. Vet. Microbiol..

[32] da Silva Ribeiro M., da Fonseca F.A.L., de Queiroz M.N., Affonso E.G., da Conceição L.E.C., Gonçalves L.U. (2017). Fish Protein Hydrolysate as an Ingredient in Diets for *Arapaima gigas* Juveniles. Bol. Inst. Pesca.

[33] Marinho R.G.B., Tavares-Dias M., Dias-Grigório M.K.R., Neves L.R., Yoshioka E.T.O., Boijink C.L., Takemoto R.M. (2013). Helminthes and Protozoan of Farmed Pirarucu (*Arapaima gigas*) in Eastern Amazon and Host-Parasite Relationship. Arq. Bras. Med. Vet. Zootec..

[34] Andrade-Porto S.M., Cárdenas M.Q., Martins M.L., Oliveira J.K.Q., Pereira J.N., Araújo C.S.O., Malta J.C.O. (2015). First Record of Larvae of *Hysterothylacium* (Nematoda: Anisakidae) with Zoonotic Potential in the Pirarucu *Arapaima gigas* (Osteichthyes: Arapaimidae) from South America. Braz. J. Biol..

[35] Proietti-Junior A.A., Lima L.S., Roges E.M., Rodrigues Y.C., Lima K.V.B., Rodrigues D.P., Tavares-Dias M. (2021). Experimental Co-Infection by *Aeromonas hydrophila* and *Aeromonas jandaei* in Pirarucu *Arapaima gigas* (Pisces: Arapaimidae). Aquac. Res..

[36] Choresca C.H., Gomez D.K., Shin S.P., Kim J.H., Han J.E., Jun J.W., Park S.C. (2011). Molecular Detection of *Edwardsiella tarda* with *gyrB* Gene Isolated from Pirarucu, *Arapaima gigas* Which Is Exhibited in an Indoor Private Commercial Aquarium. Afr. J. Biotechnol..

[37] Kodama H., Nakanishi Y., Yamamoto F., Mikami T., Izawa H., Imagawa T., Hashimoto Y., Kudo N. (1987). *Salmonella arizonae* Isolated from a Pirarucu, *Arapaima gigas* Cuvier, with Septicaemia. J. Fish. Dis..

[38] Choresca C.H., Kim J.H., Gomez D.K., Jang H., Joh S.J., Park S.C. (2008). Isolation of *Serratia fonticola* from Pirarucu *Arapaima gigas*. Korean J. Vet. Res..

[39] Barbanti A.C.C., do Rosário A.E.C., da Silva Maia C.R.M., Rocha V.P., Costa H.L., Trindade J.M., Nogueira L.F.F., Rosa J.C.C., Ranzani-Paiva M.J.T., Pilarski F. (2024). Genetic Characterization of Lactococcosis-Causing Bacteria Isolated from Brazilian Native Fish Species. Aquaculture.

[40] Serrano-Martínez E., Verónica C.P., Marco Q.H., Gina C.V., Jorge L.Q. (2014). Isolation of Bacteria and Fungi in Tissues of Paiche (*Arapaima gigas*) Reared in Captivity. Rev. Investig. Vet. Peru.

[41] Assis G.B.N., Pereira F.L., Zegarra A.U., Tavares G.C., Leal C.A., Figueiredo H.C.P. (2017). Use of MALDI-TOF Mass Spectrometry for the Fast Identification of Gram-Positive Fish Pathogens. Front. Microbiol..

[42] Poyart C., Tazi A., Réglier-Poupet H., Billoët A., Tavares N., Raymond J., Trieu-Cuot P. (2007). Multiplex PCR Assay for Rapid and Accurate Capsular Typing of Group B Streptococci. J. Clin. Microbiol..

[43] Costa F.A.A., Leal C.A.G., Leite R.C., Figueiredo H.C.P. (2014). Genotyping of *Streptococcus dysgalactiae* Strains Isolated from Nile Tilapia, *Oreochromis niloticus* (L.). J. Fish. Dis..

[44] Dice L.R. (1945). Measures of the Amount of Ecologic Association between Species. Ecology.

[45] Ishii S., Sadowsky M.J. (2009). Applications of the Rep-PCR DNA Fingerprinting Technique to Study Microbial Diversity, Ecology and Evolution. Environ. Microbiol..

[46] Hunter P.R., Gaston M.A. (1988). Numerical Index of the Discriminatory Ability of Typing Systems: An Application of Simpson’s Index of Diversity. J. Clin. Microbiol..

[47] Pinto F.R., Melo-Cristino J., Ramirez M. (2008). A Confidence Interval for the Wallace Coefficient of Concordance and Its Application to Microbial Typing Methods. PLoS ONE.

[48] Jones N., Bohnsack J.F., Takahashi S., Oliver K.A., Chan M.-S., Kunst F., Glaser P., Rusniok C., Crook D.W.M., Harding R.M. (2003). Multilocus Sequence Typing System for Group B *Streptococcus*. J. Clin. Microbiol..

[49] Jolley K.A., Bray J.E., Maiden M.C.J. (2018). Open-Access Bacterial Population Genomics: BIGSdb Software, the PubMLST.Org Website and Their Applications. Wellcome Open Res..

[50] Page A.J., De Silva N., Hunt M., Quail M.A., Parkhill J., Harris S.R., Otto T.D., Keane J.A. (2016). Robust High-Throughput Prokaryote de Novo Assembly and Improvement Pipeline for Illumina Data. Microb. genomics.

[51] Zerbino D.R., Birney E. (2008). Velvet: Algorithms for de Novo Short Read Assembly Using de Bruijn Graphs. Genome Res..

[52] Boetzer M., Henkel C.V., Jansen H.J., Butler D., Pirovano W. (2011). Scaffolding Pre-Assembled Contigs Using SSPACE. Bioinformatics.

[53] Boetzer M., Pirovano W. (2012). Toward Almost Closed Genomes with GapFiller. Genome Biol..

[54] Gurevich A., Saveliev V., Vyahhi N., Tesler G. (2013). QUAST: Quality Assessment Tool for Genome Assemblies. Bioinformatics.

[55] Seemann T. (2014). Prokka: Rapid Prokaryotic Genome Annotation. Bioinformatics.

[56] Pruitt K.D., Tatusova T., Brown G.R., Maglott D.R. (2012). NCBI Reference Sequences (RefSeq): Current Status, New Features and Genome Annotation Policy. Nucleic Acids Res..

[57] Rodrigues D.L.N., Ariute J.C., da Costa F.M.R., Benko-Iseppon A.M., Barh D., Azevedo V., Aburjaile F. (2023). PanViTa: Pan Virulence and ResisTance Analysis. Front. Bioinforma..

[58] Pritchard L., Glover R.H., Humphris S., Elphinstone J.G., Toth I.K. (2016). Genomics and Taxonomy in Diagnostics for Food Security: Soft-Rotting Enterobacterial Plant Pathogens. Anal. Methods.

[59] Emms D.M., Kelly S. (2019). OrthoFinder: Phylogenetic Orthology Inference for Comparative Genomics. Genome Biol..

[60] (2020). Methods for Antimicrobial Broth Dilution and Disk Diffusion Susceptibility Testing of Bacteria Isolated from Aquatic Animals.

[61] Leal C.A.G., Silva B.A., Colombo S.A. (2023). Susceptibility Profile and Epidemiological Cut-Off Values Are Influenced by Serotype in Fish Pathogenic *Streptococcus agalactiae*. Antibiotics.

[62] Kronvall G., Smith P. (2016). Normalized Resistance Interpretation, the NRI Method. APMIS.

[63] Magiorakos A.-P., Srinivasan A., Carey R.B., Carmeli Y., Falagas M.E., Giske C.G., Harbarth S., Hindler J.F., Kahlmeter G., Olsson-Liljequist B. (2012). Multidrug-Resistant, Extensively Drug-Resistant and Pandrug-Resistant Bacteria: An International Expert Proposal for Interim Standard Definitions for Acquired Resistance. Clin. Microbiol. Infect..

[64] Luna L.G. (1968). Manual of Histologic Staining Methods of the Armed Forces Institute of Pathology.

[65] Barony G.M., Tavares G.C., Pereira F.L., Carvalho A.F., Dorella F.A., Leal C.A.G., Figueiredo H.C.P. (2017). Large-Scale Genomic Analyses Reveal the Population Structure and Evolutionary Trends of *Streptococcus agalactiae* Strains in Brazilian Fish Farms. Sci. Rep..

[66] Laith A.A., Ambak M.A., Hassan M., Sheriff S.M., Nadirah M., Draman A.S., Wahab W., Ibrahim W.N.W., Aznan A.S., Jabar A. (2017). Molecular Identification and Histopathological Study of Natural *Streptococcus agalactiae* Infection in Hybrid Tilapia (*Oreochromis niloticus*). Vet. World.

[67] Delamare-Deboutteville J., Bowater R., Condon K., Reynolds A., Fisk A., Aviles F., Barnes A.C. (2015). Infection and Pathology in Queensland Grouper, *Epinephelus lanceolatus*, (Bloch), Caused by Exposure to *Streptococcus agalactiae* via Different Routes. J. Fish. Dis..

[68] Ortega Asencios Y., Barreiro Sánchez F., Bueno Mendizábal H., Huancaré Pusari K., Ostos Alfonso H., Manchego Sayán A., Pereira Figueiredo M.A., Gómez Manrique W., de Andrade Belo M.A., Sandoval Chaupe N. (2016). First Report of *Streptococcus agalactiae* Isolated from *Oreochromis niloticus* in Piura, Peru: Molecular Identification and Histopathological Lesions. Aquac. Rep..

[69] Gonzalez-Callirgos L., da Costa J.I., Yunis-Aguinaga J. (2023). Economic Evaluation of *Arapaima gigas* Production in Earth Ponds: Case Study of a Small Fish Farm at San Martin-Peru. Bol. Inst. Pesca.

[70] Venturieri R., Bernardino G. (1999). Pirarucu: Espécie Ameaçada Pode Ser Salva Através Do Cultivo. Panor. Aquicultura.

[71] Iregui C.A., Comas J., Vásquez G.M., Verján N. (2016). Experimental Early Pathogenesis of *Streptococcus agalactiae* Infection in Red Tilapia *Oreochromis* spp.. J. Fish. Dis..

[72] Chideroli R.T., Amoroso N., Mainardi R.M., Suphoronski S.A., de Padua S.B., Alfieri A.F., Alfieri A.A., Mosela M., Moralez A.T.P., de Oliveira A.G. (2017). Emergence of a New Multidrug-Resistant and Highly Virulent Serotype of *Streptococcus agalactiae* in Fish Farms from Brazil. Aquaculture.

[73] de Oliveira T.F., Queiroz G.A., Teixeira J.P., Figueiredo H.C.P., Leal C.A.G. (2018). Recurrent *Streptoccoccus agalactiae* Infection in Nile Tilapia (*Oreochromis niloticus*) Treated with Florfenicol. Aquaculture.

[74] Faria F.C., Leal C.A.G., Carvalho-Castro G.A., Leite R.C., Figueiredo H.C.P. (2014). Carrier State Induced by Oxytetracycline Therapy against Streptococcosis in Nile Tilapia, *Oreochromis niloticus* (L.). J. Fish. Dis..

