# Supplementary material for: Streptococcus agalactiae Infection in Wild Trahira (Hoplias malabaricus) and Farmed Arapaima (Arapaima gigas) in Brazil: An Interspecies Transmission in Aquatic Environments Shared with Nile Tilapia (Oreochromis niloticus)
Source: Microorganisms. 2024 Nov 22;12(12):2393. doi: 10.3390/microorganisms12122393 (PMC11677813; doi:10.3390/microorganisms12122393)
Supplement: Supplementary file 1 [file microorganisms-12-02393-s001.zip › microorganisms-3313952-supplementary.pdf]

Table S1- Strains, host, origin, and ST of *Streptococcus agalactiae* strains used in phylogenomic analysis

Strain	Host	State	ST	Accession Number	Reference
SA32-17	*Hoplias malabaricus*	Minas Gerais	NT		This study
SA45-17	*Arapaima gigas*	Minas Gerais	260		This study
SA1	*Oreochromis niloticus*	Minas Gerais	NT	CP019804.1	Barony et al., 2017
SA5	*Oreochromis niloticus*	Espírito Santo	NT	CP019805.1	Barony et al., 2017
SA9	*Oreochromis niloticus*	Espírito Santo	NT	CP019806.1	Barony et al., 2017
SA53	*Oreochromis niloticus*	Ceará	260	CP019802.1	Barony et al., 2017
SA73	*Oreochromis niloticus*	Ceará	260	CP019803.1	Barony et al., 2017
SA75	*Oreochromis niloticus*	Ceará	260	CP019808.1	Barony et al., 2017
SA79	*Oreochromis niloticus*	Santa Catarina	NT	CP019809.1	Barony et al., 2017
SA81	*Pseudoplatystoma* sp.	Mato Grosso	NT	CP019810.1	Barony et al., 2017
SA85	*Oreochromis niloticus*	Alagoas	927	CP019811.1	Barony et al., 2017
SA95	*Oreochromis niloticus*	Alagoas	927	CP019812.1	Barony et al., 2017
SA97	*Oreochromis niloticus*	Pernambuco	927	CP019813.1	Barony et al., 2017
SA102	*Oreochromis niloticus*	Pernambuco	927	CP019814.1	Barony et al., 2017
SA132	*Oreochromis niloticus*	Ceará	260	CP019815.1	Barony et al., 2017
SA136	*Oreochromis niloticus*	Ceará	260	CP019816.1	Barony et al., 2017
SA159	*Oreochromis niloticus*	Minas Gerais	NT	CP019817.1	Barony et al., 2017
SA184	*Oreochromis niloticus*	Espírito Santo	NT	CP019818.1	Barony et al., 2017
SA191	*Oreochromis niloticus*	Ceará	260	CP019819.1	Barony et al., 2017
SA195	*Oreochromis niloticus*	São Paulo	NT	CP019820.1	Barony et al., 2017
SA201	*Oreochromis niloticus*	Espírito Santo	NT	CP019821.1	Barony et al., 2017
SA209	*Oreochromis niloticus*	São Paulo	NT	CP019822.1	Barony et al., 2017
SA212	*Oreochromis niloticus*	São Paulo	NT	CP019823.1	Barony et al., 2017
SA218	*Oreochromis niloticus*	Espírito Santo	927	CP019824.1	Barony et al., 2017
SA220	*Oreochromis niloticus*	São Paulo	NT	CP019825.1	Barony et al., 2017
SA245	*Oreochromis niloticus*	Minas Gerais	260	CP019826.1	Barony et al., 2017
SA256	*Oreochromis niloticus*	Ceará	260	CP019827.1	Barony et al., 2017
SA289	*Oreochromis niloticus*	Ceará	260	CP019828.1	Barony et al., 2017
SA330	*Oreochromis niloticus*	São Paulo	NT	CP019829.1	Barony et al., 2017
SA333	*Oreochromis niloticus*	Goiás	NT	CP019830.1	Barony et al., 2017
SA341	*Oreochromis niloticus*	Minas Gerais	NT	CP019831.1	Barony et al., 2017
SA343	*Oreochromis niloticus*	Minas Gerais	NT	CP019832.1	Barony et al., 2017
SA346	*Oreochromis niloticus*	Minas Gerais	NT	CP019833.1	Barony et al., 2017
SA374	*Oreochromis niloticus*	São Paulo	NT	CP019834.1	Barony et al., 2017
SA375	*Oreochromis niloticus*	São Paulo	NT	CP019835.1	Barony et al., 2017
SA623	*Oreochromis niloticus*	Minas Gerais	NT	CP019836.1	Barony et al., 2017
SA627	*Oreochromis niloticus*	Minas Gerais	NT	CP019837.1	Barony et al., 2017

1. Barony, G.M.; Tavares, G.C.; Pereira, F.L.; Carvalho, A.F.; Dorella, F.A.; Leal, C.A.G.; Figueiredo, H.C.P. Large-scale genomic analyses reveal the population structure and evolutionary trends of *Streptococcus agalactiae* strains in Brazilian fish farms. *Sci. Rep.* **2017**, *7*, 13538, doi:10.1038/s41598-017-13228-z.
